# Facile synthesis and optical properties of colloidal quantum dots/ZnO composite optical resonators

**DOI:** 10.1039/c7ra12293d

**Published:** 2018-01-09

**Authors:** Jie Yu, Shaohua Dong, Hongxing Dong, Jinxin Zhan, Shulin Sun, Long Zhang

**Affiliations:** Key Laboratory of Materials for High-Power Laser, Shanghai Institute of Optics and Fine Mechanics, Chinese Academy of Science Shanghai 201800 China hongxingd@siom.ac.cn lzhang@siom.ac.cn; School of Physical Science and Technology, ShanghaiTech University 393 Middle Huaxia Road, Pudong Shanghai 201210 China; University of Chinese Academy of Sciences No. 19(A) Yuquan Road, Shijingshan District Beijing 100049 China; Department of Optical Science and Engineering and Key Laboratory of Micro and Nano Photonic Structures (Ministry of Education), Fudan University Shanghai 200433 China; IFSA Collaborative Innovation Center, Shanghai Jiao Tong University Shanghai 200240 China

## Abstract

We present a novel colloidal quantum dot (CQD)/ZnO whispering gallery mode microcavity composite. The whispering gallery mode emission of the CQDs induced by the ZnO microcavity is realized. The resonant properties of the composite optical cavities are systematically investigated, and the obtained results are supported by finite element method simulations. The work presents a new research platform to study light–matter interactions in such a composite microcavity.

## Abstract 2

We present a facile method of incorporating CdSe/Zn_*x*_Cd_1−*x*_S CQDs onto the surface of a ZnO WGM optical microcavity. The whispering gallery mode emission of the CQDs induced by the ZnO microcavity is directly observed.

## Graphical abstract

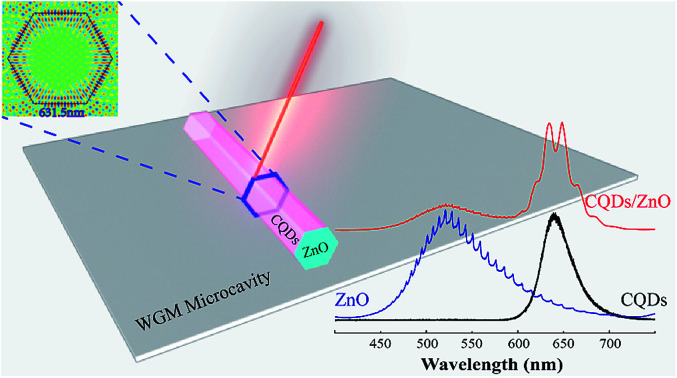

Quantum dots (QDs) feature a quantized energy structure, attracting considerable attention due to their narrow-linewidth emission spectra, high quantum efficiencies, and broad-energy-range size-tunable band gaps.^1,2^ In this research field, great efforts have been devoted to the studies of the combination of QDs with optical microcavities, which is very important both for fundamental research on light–matter interactions and for optics- and photonics-related applications. Most of the previously described composite systems feature a distributed Bragg reflector (DBR) structure and self-assembled QDs, which have allowed great progress in the development of single-photon sources,^3,4^ photodetectors,^5,6^and cavity lasers.^7,8^ However, the above QDs (used as gain materials in these composites) were mostly based on III–V semiconductors prepared by molecular beam epitaxy (MBE)^9^ or metal-organic chemical vapor deposition (MOCVD).^10,11^ Moreover, DBR-structured microcavities are usually fabricated using MBE, MOCVD, or sputtering, additionally requiring the utilization of electron beam lithography (EBL) and other nano-etching technologies.^12–16^ Thus, these sophisticated and expensive fabrication techniques and limited material availability are not conducive to the development of this research field.

In contrast, colloidal quantum dots (CQDs) exhibit the advantages of high optical stability, solution processability, and emission wavelength tunability,^17,18^ being well suited for use in composite microcavities. However, the hybridization of CQDs is difficult, with the main method used for this purpose also being rather complex, featuring the insertion of a CQDs layer into the DBR structure by spin coating.^19^ The methods like epitaxial growth *have been used* to synthesize and incorporate CQDs into a photonic crystal distributed feedback (PC-DFB) optical cavity,^20^ or fabricate on-chip microdisk laser.^21^ They all require expensive equipment, *e.g.* plasma-enhanced chemical vapor deposition (PECVD) or RF frequency sources, and the process of them are also relatively complex. In addition, alkyl modification and drop-coating also have been used to attach CQDs to silica microbeads^22^ and submicron scale grating structures^23^ showing good composite effect. Nano/microstructure optical cavities with regular geometric configurations are another important class of microcavities,^24–26^ attracting growing interest due to their ease of synthesis, high tunability, and excellent optical confinement effect. Among these cavities, ZnO microrod hexagonal whispering-gallery-mode (WGM) microcavities are the ones most extensively studied,^27–31^ allowing light confinement due to multiple total internal reflection (TIR) at resonator boundaries and thus enabling effective control of light–matter interaction. This control is essential for both fundamental physics research in the field of cavity quantum electrodynamics and the development of cavity-based optoelectronic devices, and it is therefore believed that the formation of CQDs/microcavity composites will promote further progress in the optical modulation of CQDs.

Herein, we present a facile method of incorporating CdSe/Zn_*x*_Cd_1−*x*_S CQDs onto the surface of a ZnO hexagonal microrod WGM optical cavity. The modulated emission of the CQDs induced by the ZnO microrod cavity was observed. And, the coupling properties of the CQDs/microcavity composite system have been also studied at room temperature. A whispering gallery mode (WGM) was identified by calculations based on the TIR model and further confirmed by Finite Element Method (FEM) simulations. Furthermore, the resonant properties in relation to the CQDs were studied in detail. Notably, we also demonstrate the occurrence of energy transfer between CQDs and the ZnO microcavity. Thus, our work describes a simple method of investigating optical property coupling between CQDs and nano/microstructure optical cavities.

Single-crystalline ZnO microrods were grown on a silicon (Si) substrate in a horizontal tube furnace (with no catalysts, carrier gases, low pressure, or templates used) utilizing a reduction–oxidation method similar to that described in our previous report.^32^ Core/shell CdSe/Zn_*x*_Cd_1−*x*_S CQDs were prepared as described elsewhere,^33^ purified by centrifugation and decantation using a toluene/ethanol mixture as a solvent, and redispersed in toluene. The CQDs/ZnO microrod composite was prepared by dropcasting the above dispersion onto ZnO microrods deposited on a clean Si wafer to form a thin CQDs film, with the corresponding photoluminescence (PL) spectra recorded after solvent evaporation. The morphology, composition, and microstructures of the obtained samples were characterized by field emission scanning electron microscopy (FE-SEM, Zeiss Auriga S40), high-resolution transmission electron microscopy (HRTEM, JEOL JEM-2010), and energy-dispersive spectroscopy (EDS). The optical properties of a chosen individual ZnO microrod were determined by confocal micro-photoluminescence spectrometry (JY LabRAM HR800 UV) using a 325 nm He–Cd laser as an excitation source. FEM simulations were carried out using commercial finite element software (COMSOL Multiphysics).

Fig. 1(a) shows atypical SEM image of as-synthesized ZnO microrods. A large quantity of rod-like microstructures with smooth surface was produced on the Si wafer. Most of the microrods have diameters in the range of 2–5 μm and lengths exceeding 100 μm. Microrods with smaller sizes of about several hundred nanometers were also observed. Fig. 1(b) shows the detailed morphology of a single ZnO microrod with a side length of ∼2 μm. The enlarged SEM image of the microrod exhibits a perfect hexagonal cross section and smooth surfaces, which benefit the formation of natural WGM microcavities and make such microrods ideal carriers for the study of CQDs/microcavity composites. A three-dimensional (3D) scheme of the WGM microcavity of the CQDs/ZnO microrod composite is shown in Fig. 1(c). The composite method is very simple. The CdSe/Zn_*x*_Cd_1−*x*_S core/shell semiconductor CQDs solution were dropped on the ZnO microrod. After the solvent evaporation, it then formed a thin film of CQDs. Fig. 1(d) shows the HRTEM image of the CQDs/ZnO microrod composite. It can be clearly seen that a layer of CQDs closely covering the surface of the ZnO microrod. The interface between ZnO surface and CQDs layer was labelled with a red dotted line. The CQDs are well dispersed with a diameter of about 5 nm as marked out by red circles. Meanwhile, the well-resolved lattice fringes demonstrate the highly crystallined nature of the CQDs nanocrystals. Moreover, the EDS elemental mapping further identified the presence of ZnO in the core part and of CdSe/Zn_*x*_Cd_1−*x*_S CQDs on the surface (Fig. 1(f–j)).

**Fig. 1:**
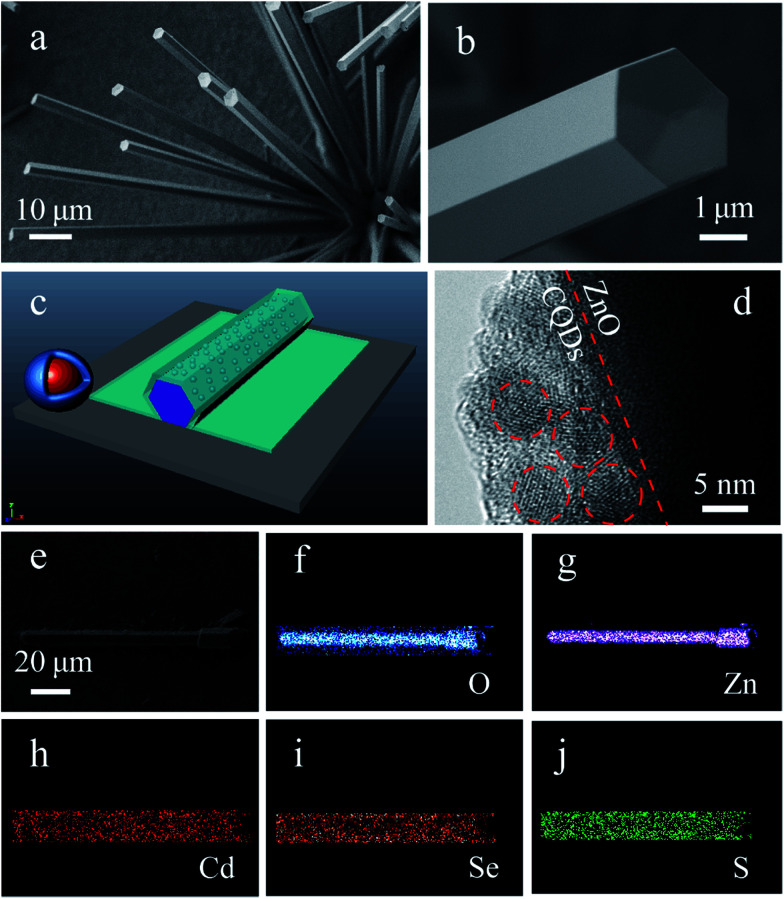
Typical SEM images of as-synthesized ZnO microrods: (a) low-magnification SEM image; (b) high-magnification SEM image revealing the morphology of an individual ZnO microrod with a hexagonal cross-section; (c) 3D scheme of a single core/shell CQDs and a CQDs/ZnO microrod composite; (d) HRTEM images of the above composite; (e) SEM image of a typical CQDs/ZnO composite; (f–j) EDS elemental mappings of O, Zn, Cd, Se, and S, respectively.

The optical properties of the individual ZnO microrod were investigated by confocal micro-photoluminescence spectrometry using an excitation laser focused by a 40× objective to a ∼2 μm spot. PL spectra were recorded using a silicon charge-coupled-device (CCD) detector and a 600 line/mm grating. Photoluminescence imaging was carried out using a self-built confocal micro-photoluminescence spectrometer with a 405 nm laser. Fig. 2(a) shows atypical PL spectrum of the ZnO microrod, revealing the presence of a characteristic ZnO exciton emission in the UV range (around 380 nm) and abroad point defect emission band between 450 and 700 nm with clear modulations. The intensity of defect emission was stronger than that of exciton emission, and the absence of obvious emission resonant modes in the UV emission band were ascribed to a mode spacing too small to be resolved in the narrow UV band, with the optical absorption around the band edge region being larger. Fig. 2(b) depicts an expanded view of resonance peaks between 480 and 590 nm, clearly showing the microrod resonator for both TE (electrical component of light *E*⊥*c*-axis) and TM (*E*∥*c*-axis) polarization configurations. From the viewpoint of geometrical optics, two kinds of resonant cavity modes may form in the microrod cavity, namely simple WGM microcavities formed by multiple TIR from the six surfaces and F–P modes formed for two pairs of opposite facets. To determine the exact mode responsible for the signal, two adjacent peaks (*λ*_1_ = 519.2 nm, *λ*_2_ = 525.9 nm) of the TM signal were selected to calculate the path length (*L*) as follows:1
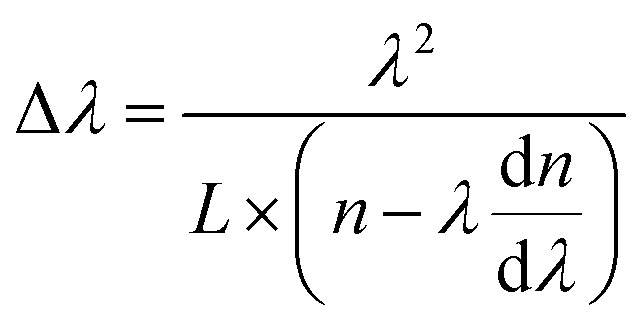
where *n* is the refractive index of the medium, and d*n*/d*λ* is the dispersion relation, with Δ*λ* (mode spacing between two adjacent peaks, also called free spectral range, FSR^34^) = 6.7 nm, *n* = 2.06 (*λ*_1_ = 519.2 nm), and *λ*d*n*/d*λ* = −0.6 obtained using the refractive dispersion of ZnO in ref. 32. The calculated path length equaled ∼15.06 μm, and the side length (*R*) of the microrod used for the PL measurement equaled 2.94 μm, as determined by SEM imaging. If the resonant modes were simple F–P modes, the deduced values of *L* would equal 4*R*, *i.e.*, ∼11.8 μm. Obviously, the calculated effective path length was much smaller than that (15.06 μm) calculated using eqn (1), which proved the above hypothesis wrong. Conversely, for the whispering gallery mode, the relevant path length was calculated as 
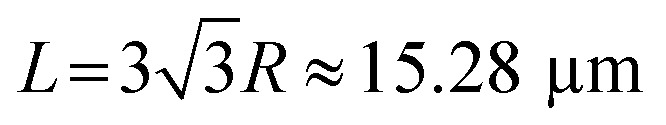
, agreeing with the theoretically calculated value given above. Thus, it was concluded that the observed resonant modes were mainly caused by the effect of the WGM microcavity. For the whispering gallery mode, the incident angle equaled 60°, with one full path featuring six TIRs. Such WGM microcavities can effectively control light emitted from ZnO itself, facilitating the research of light–matter interaction and the development of relevant optical devices.

**Fig. 2:**
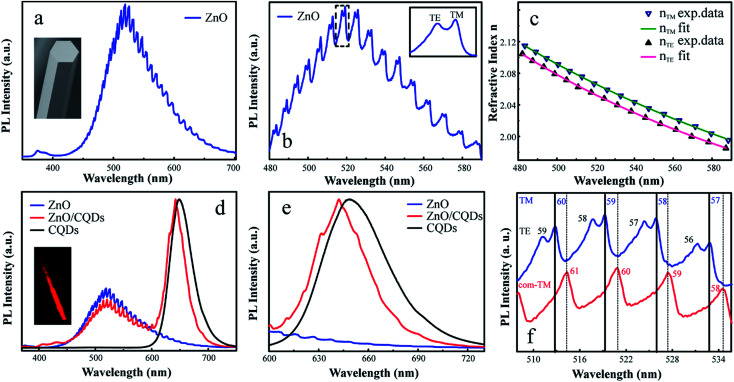
(a) PL spectrum of an individual ZnO microrod. (b) Enlarged region of the above spectrum from 480 to 590 nm. (c) Corresponding ZnO dispersion relations. (d) Full-range PL spectra of ZnO (blue line), CQDs (blackline) and CQDs/ZnO (red line), respectively; inset shows a fluorescent image of the CQDs/ZnO composite. (e) The PL spectrum of CQDs in the range of 600–725 nm. (f) Corresponding resonator mode numbers of pure ZnO and the CQDs/ZnO composite in the range of 508–536 nm.

To further explore the characteristics of the ZnO microrod WGM resonator, we identified the interference order *N* for TE and TM modes using the following equation:2
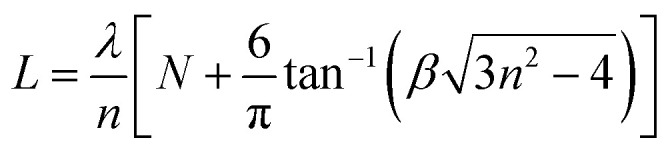
where *n* is the refractive index of the ZnO sample, and *N* is the interference order of the resonant mode. The factor *β* is dependent on polarization. For TE polarization *β* = *n*, for TM polarization *β* = 1/*n*.

The interference order *N* of the TE and TM modes were initially identified, using the refractive dispersion of ZnO microtubes.^26^ The best fit of the interference order (*N*_TE_ = 47–66, *N*_TM_ = 48–67) was obtained by varying *N* systematically and the cavity length *L* within the experimental error. A similar fitting process has been utilized to calculate the refractive indices of ZnO microtubes.^35^ These two series of integers are the interference orders for the relevant resonant modes between 480 nm and 590 nm. Using the obtained interference orders and the cavity length *L*, the accurate wavelength–dependent refractive dispersions (*n*_TE_ & *n*_TM_) of the ZnO microrod were calculated. The dispersion relation is shown in Fig. 2(c), and the fitting Cauchy dispersion formula as follows:3
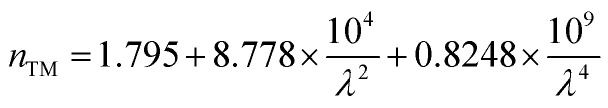
4
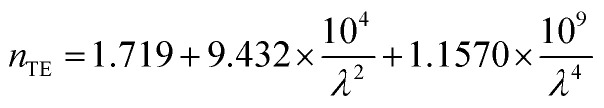


It is worth noting that at a given wavelength, *n*_TM_ is larger than *n*_TE_, with both indices decreasing with increasing wavelength.

The formation of a CQDs/ZnO microrod WGM microcavity composite was confirmed by fluorescence imaging, which revealed that the ZnO microrod cavity was decorated with CQDs emitting red light with a wavelength of ∼650 nm (inset in Fig. 2(d)). To further elucidate the optical performance of the composite, we compared it with the PL spectrum of the pure CQDs and ZnO together. Interestingly, we found that some resonant peaks appear in the CQDs emission region in the CQDs/ZnO composite system. This indicates that the light of CQDs may be introduced into the ZnO microcavity and then was modulated. In fact, the thickness of the combined CQDs layer on the surface of the microcavity is critical for the optical resonance of the CQDs. If the combined CQDs layer was too thick, it will weaken the modulated light coupled in the microcavity emitted out. This phenomenon was also observed in other composite system.^28^ In addition, it is worth noting that the CQDs emission was clearly blue-shifted after hybridization with the microcavity (Fig. 2(e)), probably due the formation of an oxidized layer on the CQDs surface under ambient conditions,^28^ which also decreased the effective CQDs size. Fig. 2(f) shows an expanded view of resonance peaks between 508 and 536 nm, demonstrating that the resonant modes of the CQDs/ZnO microrod cavity were preferentially TM-polarized and clearly red-shifted, with TE modes being very weak and difficult to observe. This behavior was ascribed to the refractive index change of the medium caused by CQDs hybridization, as described by the following formula:^36^5
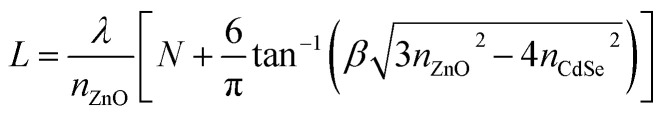
where *n*_CdSe_ is the refractive index of CQDs. The refractive index^37^ of CQDs is *n*_CdSe_ = 1.73, which is larger than that of the air medium. For the same resonant peak, the wavelength of the resonant peak will increase with the decrease of the relative difference of the refractive index, resulting in a redshift as shown in Fig. 2(f). Moreover, the deposition of a CQDs layer on the surface of the microrod cavity mainly increases the optical loss of the TE polarization mode, complicating its detection.

Interestingly, we also noticed that a broad and weak emission in the CQDs/ZnO composite microcavity appears obviously from 400 to 440 nm as shown in Fig. 3(a). And, the intensity of the exciton emission (from 370 to 390 nm) of ZnO decreases. In addition, we found that the CQDs used in our experiments also have a broad and weak emission at the same wavelength band as shown in Fig. 3(b). Moreover, the shape of the emission peak is very similar to that of the composite microcavity at the same region. This indicates there may be energy transfer between the ZnO and the combined CQDs. In fact, the broad weak emission was attributed to CdS CQDs, which synthesized along with the synthesis process of the core/shell CdSe/Zn_*x*_Cd_1−*x*_S CQDs. From Fig. 3(c), it is clearly seen that the absorption spectrum of CdS CQDs covers the emission wavelength of the ZnO excitons at ∼390 nm. The central emission wavelength of CdS CQDs is ∼406 nm, and full width at half-maximum is 25 nm. After the CQDs attached to the surface of ZnO microrod, the distance between CQDs and microrod is close enough for fluorescence resonance energy transfer (FRET) to occur. The inset of Fig. 3(c) is the energy band structure of ZnO and CdS CQDs.^2,38^ During FRET, the exciton of ZnO, initially in its electronic excited state, transfers its energy to the acceptor CdS *via* non-radiative dipole–dipole coupling, damping the band gap emission of the ZnO microrod and enhancing the emission intensity of CdS CQDs, which explains the PL spectra shown in Fig. 3(a). However, the above behavior was not observed when a purified CQDs solution (free of CdS CQDs) was used under the same experimental conditions (Fig. 3(d)), further verifying the occurrence of FRET in the CQDs/ZnO composite microcavity.

**Fig. 3:**
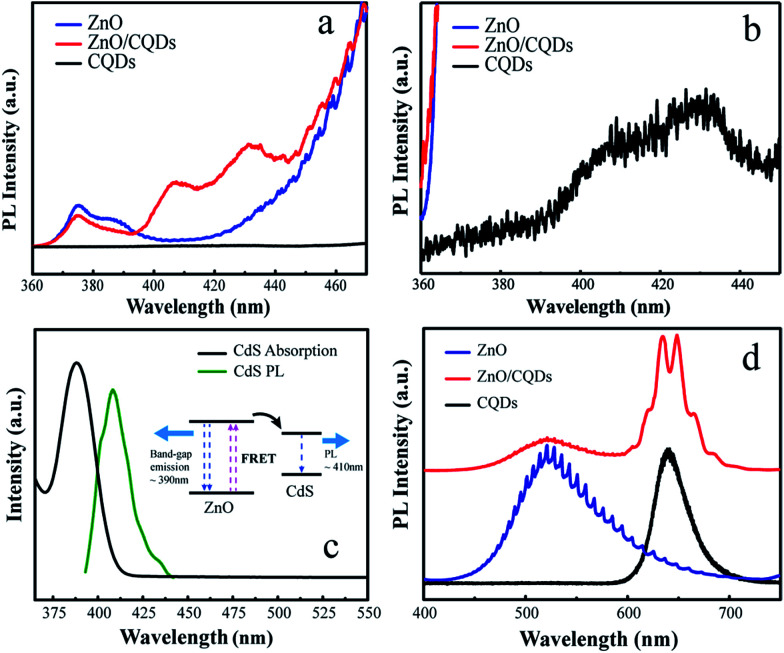
(a) PL spectra expanded in the range of 360–470 nm. (b) PL spectrum of CQDs in the range of 360–450 nm. (c) Absorption and PL spectra of CdS CQDs, with inset showing a FRET diagram with typical timescales. (d) PL spectra of CdS CQDs–free CdSe/Zn_*x*_Cd_1−*x*_S core/shell CQDs, ZnO microrods, and the CQDs/ZnO composite.

To clarify the nature of the resonance modes observed in the PL measurements, FEM simulations are used to study such ZnO or CQDs/ZnO microrod composite microcavity with hexagonal cross-section. Because of the two-dimensional (2D) nature of the measured optical modes, we only simulate a 2D model to simplify our calculation. In our simulations, the modeled microcavity with the same hexagonal cross-section as the fabricated microrod shown in Fig. 1(a) (*i.e.*, a side length of 2.94 μm) is placed inside a simulation box surrounded by the well-matched layer boundaries to absorb the scattered electromagnetic fields. Here, TM polarization was chosen for comparing experimental and simulated results, and the dispersive refraction index of ZnO was therefore calculated from the PL spectrum of this material using eqn (3). The refractive index of CQDs was assumed to equal 1.73. And the dispersive refractive index of ZnO described by eqn (3) is directly imported into the software. The background medium in the simulation box was set to air or CdSe for investigating the microcavities of ZnO or the CQDs/ZnO composite, respectively. An electric current source was placed inside the 2D microcavity to excite TM-polarized optical modes. We choose a very dense mesh inside the ZnO microrod (<*λ*/20) and surrounding air (<*λ*/10) to guarantee the convergence of our results.

The calculated radiation intensity spectra of the current source inside ZnO and CQDs/ZnO composite microcavities are shown in Fig. 4(b and d), respectively, revealing that if the radiation wavelength of the line source matches that of a microcavity resonance mode, its radiation is significantly enhanced, with these peaks being unambiguous signatures of the optical modes excited in the microcavity. In our calculation, the intensity of the current source was identical for all excitation wavelengths and, therefore, key information was provided only by the position of radiation peaks, with its intensity being negligible. For both ZnO and CQDs/ZnO composite microcavities, the resonance peaks of FEM-simulated radiation spectra well matched those observed experimentally (Fig. 4(a–d)), with the slight mismatch observed for CQDs/ZnO at short wavelengths attributed to the slight poor dispersion of CdSe that was ignored in our simulation (Fig. 4(c and d)). If the microcavity is surrounded by CQDs instead of the air, the resonance modes leak out of the ZnO microcavity more easily owing to the increased refraction index of the background medium, which increases the effective optical path for the resonance modes and induces their red shift (Fig. 4(b and d)). To justify these arguments, we further utilized the eigenmode analysis solver of COMSOL Multiphysics to search all eigen resonance modes supported by the two microcavities. For example, Fig. 4(e–h) show the electric field patterns of two representative resonance modes, clearly identifying the features of WGMs with *N* = 47 and 55. Likewise, the calculated resonance wavelengths (see insets) perfectly matched the peak positions marked by dashed lines in Fig. 4(a–d), demonstrating that *N* = 55 (*N* = 47) resonance modes shift from 547.8 nm (618.0 nm) in the ZnO microcavity to 556.9 nm (631.5 nm) in the CQDs/ZnO composite microcavity. In particular, the long-wavelength modes located in the fluorescence region of CdSe can indeed modulate the light emission of CQDs.

**Fig. 4:**
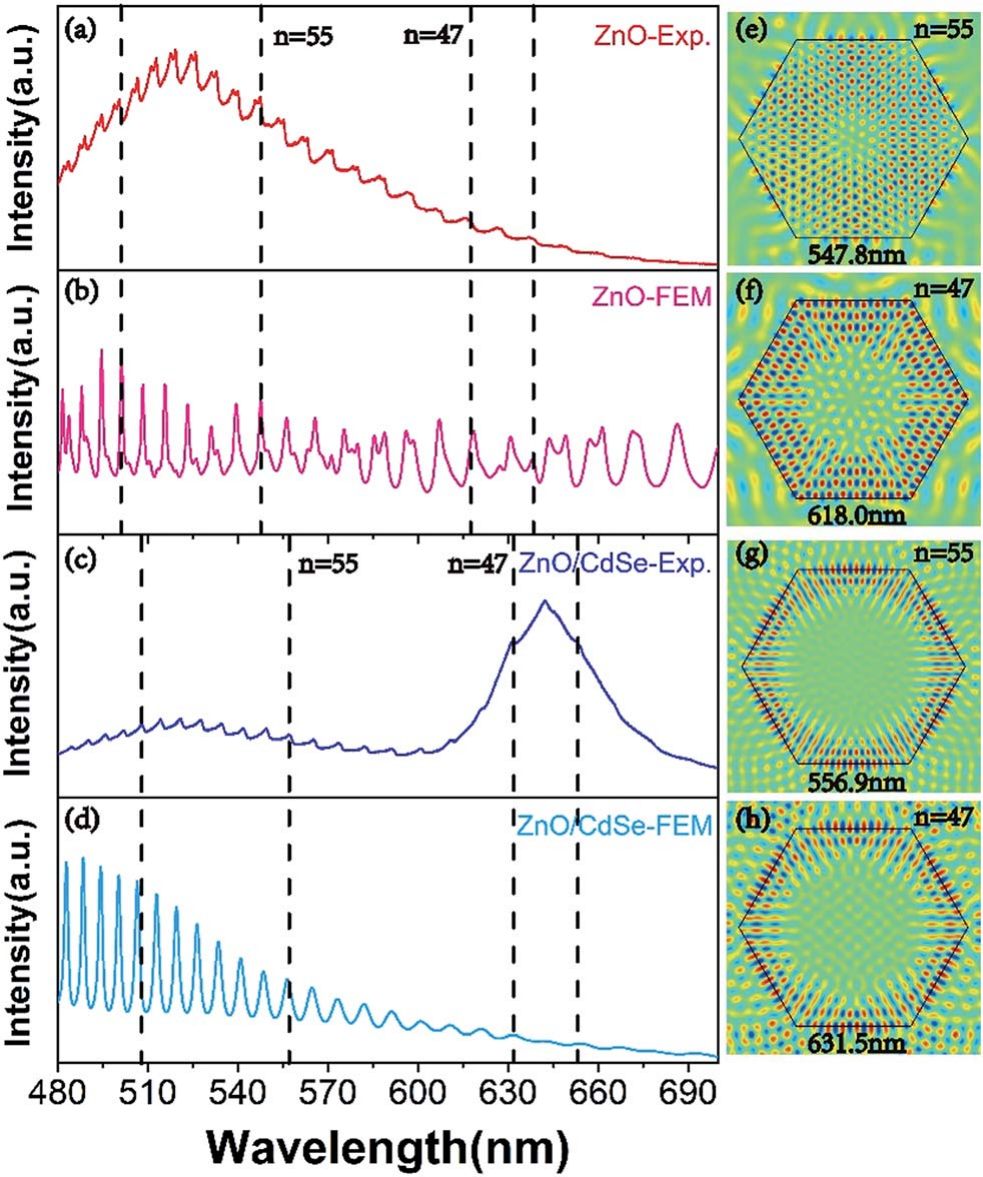
Measured PL spectra (a, c) and FEM-simulated radiation spectra (b, d) of a single ZnO microrod (a, b) and a CQDs/ZnO composite (c, d) with a hexagonal cross-section, and the corresponding WGM electric field distributions (e–h) at specified wavelengths.

## Conclusions

In summary, we have developed a simple approach for the incorporation of the CQDs on the surface of hexagonal microrod WGM microcavity. Whispering gallery mode emission of the CQDs induced by the ZnO microcavity were directly observed at room temperature. Theoretical analyses based on plane plane-wave model and FEM simulations were in good agreement with experimental results. The effect of CQDs hybridization on light modulation was discussed in detail, and the CQDs–microcavity energy transfer was investigated. Our work demonstrates that such composite microcavities provide a new research platform for studying light–matter interaction and afford CQDs/microcavity composites with increased tunability.

## Conflicts of interest

There are no conflicts to declare.

## Supplementary Material
